# Thrombosed Giant Multifocal Hepatic Cavernous Hemangioma Mimicking Lymphatic Malformation on Coaxial Biopsy in an Adult with Recurrent Fever: A Case Report and Focused Literature Review

**DOI:** 10.3390/jcm15197366

**Published:** 2026-09-22

**Authors:** Hao Cheng, Jiaxi Ma, Jinghang Xu, Li Liang, Ke Wang, Guiqiang Wang, Na Huo, Yan Wang

**Affiliations:** 1 Department of Infectious Diseases, Peking University First Hospital, Beijing 100034, China; 2 Department of Pathology, Peking University First Hospital, Beijing 100034, China; 3 Department of Medical Imaging, Peking University First Hospital, Beijing 100191, China

**Keywords:** hepatic cavernous hemangioma, hepatic lymphatic malformation, thrombosed hemangioma, recurrent fever, multiple cystic hepatic lesions, liver biopsy

## Abstract

Hepatic cavernous hemangioma is the most common benign liver tumor, but giant or extensively thrombosed variants may lose their characteristic imaging features and mimic other cystic or vascular lesions. A 32-year-old man presented with recurrent fever for more than two months, massive hepatomegaly, leukocytosis (white blood cell count 14.46 × 10^9^/L), C-reactive protein 165.30 mg/L, and greatly elevated D-dimer (48.44 mg/L) and fibrin degradation products (323.52 mg/L), with normal platelet count, fibrinogen, prothrombin time, and activated partial thromboplastin time. Computed tomography (CT) and magnetic resonance imaging (MRI) demonstrated multiple bilateral cystic or cystic-solid hepatic lesions with calcifications and no classic blood-pool enhancement pattern. ^18^F-fluorodeoxyglucose positron-emission tomography/computed tomography (PET/CT) showed no significant uptake, and an extensive microbiological workup was negative. Ultrasound-guided percutaneous biopsy (coaxial technique, 15-gauge needle, five cores from the solid component of the largest right-lobe lesion) favored a benign vascular/lymphatic tumor most consistent with hepatic lymphatic malformation (LM). Subsequently, the patient underwent staged laparoscopic partial hepatectomy combined with microwave ablation at another hospital. The resection pathology demonstrated cystically dilated, blood-filled channels with organizing thrombi, infarction, cholesterol granulomas, and calcification, diagnostic of hepatic cavernous hemangioma with thrombus formation—discordant with the biopsy interpretation. The patient has remained afebrile since surgery, with normalization of inflammatory markers and a fall in D-dimer to 6.95 mg/L. Extensive thrombosis, fibrosis, and calcification can abolish the classic imaging and histopathological features of hepatic hemangioma and make it resemble a lymphatic malformation on coaxial biopsy.

## 1. Introduction

Hepatic cavernous hemangioma is the most common benign liver tumor, with a prevalence of 3–20% in autopsy and imaging series [1,2]. Classic hemangiomas are readily diagnosed by their characteristic MRI appearance—markedly T2-hyperintense with peripheral nodular discontinuous enhancement and progressive centripetal fill-in—and generally require no tissue confirmation [2]. Giant hemangiomas (conventionally defined as >5 or >10 cm), however, may undergo internal thrombosis, infarction, sclerosis, and calcification over time. These involutional changes can abolish the diagnostic blood-pool enhancement pattern, producing heterogeneous, non-enhancing, or cystic-appearing lesions that overlap radiologically with hepatic abscesses, echinococcosis, mucinous cystic neoplasms, cystic metastases, and even lymphatic malformations (LM) [2,3].

Coaxial biopsy is sometimes performed when imaging is inconclusive and malignancy or infection cannot be excluded. In thrombosed or sclerosed hemangiomas, however, the sampled tissue may consist predominantly of fibrous septa, organizing thrombus, and collagenized stroma rather than the characteristic dilated, blood-filled cavernous spaces, further obscuring the diagnosis. Focal or partial expression of the lymphatic marker D2-40 (podoplanin) can occur in non-lymphatic vascular lesions [4], adding an additional source of misinterpretation in small biopsy samples.

Occasionally, giant hepatic hemangiomas with extensive thrombosis present with systemic symptoms such as fever of unknown origin, marked inflammatory activation, or localized intravascular coagulopathy with very high D-dimer and fibrin degradation products—a Kasabach-Merritt-like phenomenon [5,6,7,8,9]. Combined with atypical imaging, these features may suggest infection or malignancy and lead to a prolonged workup.

We report a 32-year-old man with recurrent fever, massive hepatomegaly, and multifocal hepatic cystic-solid lesions in whom coaxial biopsy first favored hepatic LM, whereas the later resection pathology established a diagnosis of cavernous hemangioma with extensive thrombosis. The case shows how coaxial biopsy can mislead in large, heterogeneous, thrombosed vascular lesions, and why clinicoradiologic–pathologic correlation and, where available, resection pathology remain the basis for the final diagnosis.

## 2. Case Presentation

A 32-year-old man was admitted to the Department of Infectious Diseases, Peking University First Hospital on 30 December 2025, for recurrent fever of more than two months. In early October, he developed a 10-day episode of low-grade fever (maximum, 38.3 °C) and dry cough. A local hospital reportedly diagnosed Mycoplasma infection, although the original report was unavailable. Five days of intravenous azithromycin followed by approximately one week of cephalosporin therapy were associated with temporary defervescence. A similar febrile episode recurred in November and again improved after azithromycin. On 24 December, fever recurred, reaching 38.5 °C, with cough but without chills, productive sputum, myalgia, abdominal pain, dyspnea, weight loss, or altered bowel habits.

On 27 December, laboratory testing at a local hospital showed a white blood cell count of 11.0 × 10^9^/L, neutrophils of 77%, and C-reactive protein (CRP) of 135 mg/L. Chest CT identified a 4 × 3-mm right upper-lobe pulmonary nodule. Contrast-enhanced abdominopelvic CT demonstrated an irregularly enlarged liver containing multiple well-defined, round or lobulated low-attenuation lesions. Several lesions contained punctate calcifications, some projected beyond the hepatic contour, and the largest measured approximately 81 × 113 mm. Definite enhancement was not reported, and the intrahepatic and extrahepatic bile ducts were not dilated. Oral levofloxacin was initiated.

At our outpatient clinic on 28 December, the maximum temperature was 39.8 °C. The white blood cell count was 14.46 × 10^9^/L, hemoglobin 105 g/L, platelet count 207 × 10^9^/L, and CRP 165.30 mg/L. Alanine and aspartate aminotransferases were normal, whereas alkaline phosphatase (138 IU/L), gamma-glutamyl transferase (188 IU/L), direct bilirubin (10.30 μmol/L), and total bile acids (11.78 μmol/L) were mildly elevated. Prothrombin time was 13.6 s, activated partial thromboplastin time 29.5 s, and fibrinogen 2.25 g/L (all within the normal range). The international normalized ratio was 1.23, D-dimer was 48.44 mg/L (DDU), and fibrin degradation products were 323.52 mg/L. Peripheral-blood targeted next-generation sequencing and Mycoplasma pneumoniae IgM were negative. On admission, he was hemodynamically stable. The liver was firm, irregular, non-tender, and palpable to the level of the umbilicus and nearly to the pelvis. He had no jaundice, peripheral edema, superficial lymphadenopathy, or peritoneal signs. His medical and family histories were unremarkable; he reported long-term smoking, regular alcohol consumption, recent dog ownership, and no relevant endemic-water or endemic-area exposure.

The initial differential diagnosis included hepatic abscesses, echinococcosis or other parasitic infection, tuberculosis, cystic or mucinous neoplasm, metastatic disease, hemangioma, and epithelioid hemangioendothelioma. Blood cultures, T-SPOT.TB, purified protein derivative testing, serologic tests for Echinococcus, Clonorchis, Taenia solium, and sparganosis, peripheral-blood targeted next-generation sequencing, and subsequent liver-tissue targeted next-generation sequencing were negative. Procalcitonin was only mildly elevated and decreased from 0.32 to 0.08–0.10 ng/mL during hospitalization; interleukin-6 was 35.12 pg/mL at admission. Tumor markers and upper and lower gastrointestinal endoscopy did not identify a primary malignancy. Duplex ultrasonography of the lower extremities showed no venous thrombosis. Empirical meropenem was administered initially; however, fever continued intermittently and inflammatory markers showed no sustained antibiotic-associated improvement. Meropenem was discontinued on 9 January, after which the temperature and inflammatory indices continued to decrease, further reducing the likelihood of an active bacterial infection. The clinical course and major diagnostic and therapeutic events are summarized in Table 1.

PET/CT on 4 January demonstrated multiple low-attenuation hepatic lesions with calcifications but no significant increase in FDG uptake. The small pulmonary nodule also showed no increased uptake, and no hypermetabolic primary tumor was identified. Contrast-enhanced MRI on 6 January showed multiple cystic hepatic lesions with possible calcifications; atypical infection and neoplasm remained in the differential diagnosis (Figure 1). A multidisciplinary conference on 12 January concluded that the multifocal, closely apposed lesions would be difficult to resect safely at that stage and recommended tissue diagnosis. On 14 January, ultrasound-guided percutaneous coaxial biopsy with tract ablation was performed. The procedure was uncomplicated, tissue targeted next-generation sequencing was negative, and follow-up ultrasound showed no intrahepatic hematoma or subcapsular hemorrhage. The fragmented cores measured 2.2 cm in aggregate and contained approximately six incomplete portal tracts. Histology showed expanded portal areas containing variably dilated vascular/lymphatic channels with focally uneven wall thickness, lined by flattened endothelial cells without significant cytologic atypia, together with stromal fibrosis and collagenization. Some enlarged portal areas contained lymphocytes, plasma cells, neutrophils, and ductular proliferation (Figure 2). CD31 and CD34 highlighted vascular structures; p53 showed a wild-type pattern and the Ki-67 labeling index was 2–3%. ERG was negative in the initial report but showed partial nuclear positivity on supplemental review, and D2-40 showed partial membranous positivity. Reticulin staining demonstrated residual hepatic plates, and Masson trichrome highlighted collagen deposition. The final report favored a benign vascular/lymphatic tumor. In conjunction with the clinicoradiologic findings, the lesion was considered most consistent with hepatic LM (Figure 3).

By 15 January, the white blood cell count had decreased to 8.60 × 10^9^/L and CRP to 63.75 mg/L. The maximum recorded temperature before discharge was 37.3 °C. He had no abdominal pain, post-biopsy fever, gastrointestinal bleeding, or hemodynamic instability and was discharged on 16 January with instructions to avoid activities that could increase the risk of hepatic rupture and to follow up with infectious-disease and hepatobiliary-surgery services.

With the patient’s consent, the complete operative record and final resection pathology were subsequently obtained from the hospital where he underwent surgery. On 31 March 2026, approximately two and a half months after discharge from our hospital, the patient underwent laparoscopic partial hepatectomy combined with intraoperative microwave ablation as the first stage of a staged treatment strategy for multifocal bilobar disease. Laparoscopic exploration confirmed multiple vascular-origin space-occupying lesions distributed in both hepatic lobes; several lesions protruded beyond the hepatic surface with tense, thin walls and a rich blood supply. The largest lesion, located in segment VI and measuring approximately 10 cm in diameter, protruded into the peritoneal cavity and compressed the hepatic flexure of the colon; a segment IVb lesion protruded from the hepatic surface and compressed the gallbladder. Two exophytic right-lobe surface lesions were treated with microwave ablation, and the segment VI and segment IVb lesions were resected along a thermally precoagulated transection line.

The resection pathology (largest specimen 9.7 × 6.1 × 4.2 cm, with a cystic-solid cut surface and cyst cavities of 0.2–1.3 cm) demonstrated a vascular-origin tumor with cystically dilated channels lined by endothelium without significant cytologic atypia, intraluminal hemorrhage with thrombus formation, stromal fibrous proliferation with infarction, hyalinization and abundant calcium deposition, and cholesterol clefts with a multinucleated giant-cell reaction, together with a mild lymphoplasmacytic infiltrate. The diagnosis was hepatic cavernous hemangioma with thrombus formation. The cauterized resection margin could not be confirmed to be free of lesion, and clinical follow-up for recurrence was recommended.

The patient has remained afebrile since the operation. Recent follow-up laboratory tests showed a white blood cell count of 4.94 × 10^9^/L, hemoglobin 153 g/L, platelet count 213 × 10^9^/L, alanine aminotransferase 20.1 IU/L, aspartate aminotransferase 19.5 IU/L, total bilirubin 12.9 μmol/L, albumin 44 g/L, prothrombin time 10.8 s, activated partial thromboplastin time 28.3 s, and fibrinogen 2.3 g/L. D-dimer had fallen from 48.44 mg/L at presentation to 6.95 mg/L. The patient remains under surveillance, and further staged treatment of the residual lesions is planned.

On the basis of the resection pathology—which demonstrated organizing thrombi, infarction, and a cholesterol-granulomatous giant-cell reaction—and the postoperative course—with sustained defervescence, normalization of the white blood cell count and inflammatory markers, and a marked decline in D-dimer—the recurrent fever and the inflammatory and coagulation abnormalities were ultimately attributed to sterile inflammation and localized intravascular coagulation arising within the extensively thrombosed and partly infarcted hemangiomas. Although a definite causal relationship cannot be proven in a single case, the resolution of all symptoms and laboratory abnormalities after surgical treatment strongly supports this interpretation.

## 3. Discussion

This patient presented with recurrent fever, inflammatory and coagulation-system activation, massive hepatomegaly, and multifocal calcified cystic or cystic-solid hepatic lesions. The diagnostic process initially centered on infection and malignancy, and the core-biopsy interpretation favored hepatic LM, whereas the resection pathology obtained later established a diagnosis of cavernous hemangioma with extensive thrombosis.

Several factors converged to make the biopsy misleading. The needle targeted the solid component of the lesion, which was the region most altered by thrombosis and fibrosis, so the fragmented cores sampled fibrous septa, collagenized stroma, and organizing thrombus rather than the dilated, blood-filled cavernous spaces that would have identified a hemangioma. On these cores, D2-40 showed partial membranous positivity in thin-walled endothelium-lined channels without visible blood-pool content, and this was read as supporting lymphatic differentiation; yet focal or partial D2-40 expression is not specific for LM and occurs in non-lymphatic vascular lesions, inflammatory conditions, and reactive endothelium [4]. The panel was also incomplete, as PROX1 and VEGFR3, which are more specific for lymphatic endothelium than D2-40 [4], were not performed. Finally, the classic imaging features of hemangioma—peripheral nodular discontinuous enhancement with centripetal fill-in—were absent, so imaging offered no correction to the histological reading. Together these features illustrate the sampling limitation of coaxial biopsy in large, heterogeneous, thrombosed vascular lesions, in which fibrotic septa and organizing thrombus may dominate the tissue obtained.

The pathological differential diagnosis of multifocal cystic or cystic-solid hepatic lesions with bland endothelium includes several entities. (1) Cavernous hemangioma versus LM: both are lined by bland, flattened endothelium; the distinction rests on the luminal content (blood and organizing thrombus versus proteinaceous lymph), the stromal changes, and lymphatic-marker expression (D2-40, PROX1, VEGFR3). In the resection specimen, blood-filled cystically dilated channels, organizing thrombi, infarction, and cholesterol granulomas were diagnostic of cavernous hemangioma with thrombosis (Table 2). (2) Biliary hamartoma (von Meyenburg complex) was excluded by the absence of small, irregular, keratin-positive biliary ductal structures embedded in fibrous stroma. (3) Mucinous cystic neoplasm and other cystic biliary neoplasms were excluded by the absence of mucinous columnar epithelium and ovarian-type subepithelial stroma. (4) Epithelioid hemangioendothelioma was excluded by the absence of epithelioid endothelial cells with intracytoplasmic lumina in a myxohyaline stroma, together with the very low Ki-67 labeling index (2–3%). (5) Angiosarcoma was excluded by the absence of endothelial multilayering, cytologic atypia, mitotic activity, and infiltrative sinusoidal growth, and by the wild-type p53 pattern. (6) Inflammatory pseudotumor-like lesions were excluded by the absence of a dominant myofibroblastic spindle-cell proliferation and of a dense IgG4-positive plasma-cell infiltrate.

The imaging findings were diagnostically challenging. Ultrasonography characterized the lesions as predominantly solid and raised concern for metastases or epithelioid hemangioendothelioma, whereas CT and MRI emphasized their low-attenuation or fluid-signal, septated, multicystic nature. Calcification, exophytic growth, and variable wall or septal enhancement further broadened the differential diagnosis. Low FDG uptake argued against many aggressive malignancies but could not distinguish hemangioma from LM, infection, or low-grade neoplasia. The radiological differential diagnosis of multifocal cystic or cystic-solid hepatic lesions, including the classic and thrombosed variants of hemangioma, is summarized in Table S1. In the present case, the absence of the classic blood-pool enhancement pattern, explained retrospectively by extensive thrombosis, fibrosis, and calcification, was the key reason the hemangioma was not recognized preoperatively on imaging.

Fever is an unusual presenting feature of hepatic hemangioma, but several adult cases of giant hepatic hemangioma with fever of unknown origin, marked inflammatory activation, or both have been reported (Table S2). The pathological substrate appears to be intralesional necrosis, hemorrhage, or organizing thrombosis, generating a sterile inflammatory response that can produce recurrent fever, leukocytosis, and elevated CRP, erythrocyte sedimentation rate, and IL-6 [5,6,7]. In the present case, the resection specimen showed organizing thrombi, stromal infarction, and cholesterol clefts with a multinucleated giant-cell reaction—direct histological evidence of ongoing intralesional tissue injury and a granulomatous, foreign-body-type inflammatory response. The patient has remained afebrile since the operation, paralleling the postoperative defervescence described by Wang et al. [6] and Ryozawa et al. [7]. Although a definite causal relationship cannot be formally proven in a single case, the convergence of histological evidence and clinical response makes sterile intralesional inflammation the most plausible mechanism.

The very high D-dimer (48.44 mg/L) and FDP (323.52 mg/L) draw our attention. Giant hepatic hemangiomas with intralesional thrombosis are a recognized cause of localized intravascular coagulopathy, in which ongoing intratumoral clot formation and secondary fibrinolysis raise D-dimer and FDP [8,9]. In extensive cases, an overt Kasabach–Merritt-like consumptive coagulopathy with thrombocytopenia and hypofibrinogenemia has been reported [8,9,10]. In our patient, the platelet count (207 × 10^9^/L at admission) and fibrinogen (2.25 g/L) remained normal, and prothrombin time (13.6 s) and activated partial thromboplastin time (29.5 s) were normal, pointing to a localized rather than a systemic consumptive process. Duplex ultrasonography of the lower extremities excluded deep-vein thrombosis, and no portal or hepatic vein thrombosis was seen on contrast-enhanced imaging. Malignancy was excluded by PET/CT, endoscopy, and finally by the resection pathology, and the clinical course argued against infection. After surgery, D-dimer fell to 6.95 mg/L, consistent with an intralesional origin. Multifocal bilobar giant hemangiomas with recurrent fever and this degree of D-dimer and FDP elevation, but without overt consumptive coagulopathy, appear to be rare in the published literature (Table S2).

Although the biopsy did not reach the correct specific diagnosis, it was far from unhelpful. It gave clear negative evidence—no malignancy, no granulomatous infection, and negative tissue tNGS—which allowed empirical antimicrobial therapy to be stopped and prevented further escalation. It also established the benign vascular nature of the lesions, with no cytologic atypia, a low Ki-67 index, and a wild-type p53 pattern, and this supported a plan of elective staged surgery rather than urgent oncologic treatment. The discordance between the biopsy (favoring LM) and the resection pathology (hemangioma with thrombosis) reflects a familiar problem, as follows: in a thrombosed, fibrotic vascular lesion, the fragmented cores may capture only the remodeled stroma rather than representative cavernous spaces, and the reading then rests on an immunophenotype that was incompletely characterized. Coaxial biopsy can narrow the differential and guide management, but its sampling limitations should be kept in view, and the definitive diagnosis should come from resection pathology when surgery is performed.

Reported adult cases of giant hepatic hemangioma presenting with fever, marked inflammation, or Kasabach–Merritt-like coagulopathy are summarized in Table S2. Lee et al. [5] reported fever of unknown origin in a patient with giant hepatic hemangioma. Wang et al. [6] described a 59-year-old woman with a 10 cm solitary hemangioma who presented with fever up to 40 °C for six months, night sweats, and weight loss; the resection specimen showed hemorrhage, necrosis, and inflammatory infiltration, and fever resolved postoperatively. Ryozawa et al. [7] reported a postpartum woman with a 13 cm right-lobe hemangioma and fever of unknown origin, in whom the resection pathology showed coagulative necrosis and neutrophilic infiltration and fever resolved after hepatectomy. Aslan et al. [8] and Deng et al. [10] reported classic Kasabach–Merritt syndrome with thrombocytopenia and consumptive coagulopathy in giant hepatic hemangioma, and Shah et al. [9] described subclinical Kasabach–Merritt phenomenon with mild thrombocytopenia and prolonged prothrombin time. Our patient’s presentation shares features of both the febrile and the coagulopathic phenotypes, yet was distinctive in that the D-dimer and FDP elevations were extreme while platelet count and fibrinogen remained normal.

The management of symptomatic or diagnostically uncertain multifocal bilobar hepatic hemangiomas is challenging and must be individualized. Options include the following: observation for asymptomatic stable lesions; enucleation or resection of dominant symptomatic or diagnostically uncertain lesions; staged resection and debulking when complete one-stage clearance is not feasible; thermal ablation (microwave or radiofrequency) as an adjunct or alternative for smaller or deep lesions; transarterial embolization for vascular supply reduction; and, in exceptional diffuse cases, liver transplantation [11,12]. For lymphatic malformations, sclerotherapy for macrocystic disease and systemic mTOR-pathway inhibition (sirolimus) for refractory or diffuse disease are additional options [13,14]. The treatment received by our patient—staged laparoscopic resection of the two dominant lesions (segments VI and IVb) combined with microwave ablation of smaller exophytic surface lesions—illustrates an individualized, parenchyma-sparing approach, and the incompletely cauterized margin and residual lesions mandate continued imaging surveillance.

The clinical lesson is that extensive thrombosis, fibrosis, and calcification can turn a common benign tumor into an imaging and histopathological mimic of a much rarer entity. When coaxial biopsy of a large, heterogeneous, vascular lesion suggests a diagnosis that does not fully explain the clinical picture, the small tissue volume and its sampling limitations should be weighed, and resection pathology sought for confirmation when surgery is undertaken.

## 4. Limitations

This report has several limitations. First, the sampling bias inherent to coaxial biopsy of large heterogeneous lesions led to an initially erroneous favored diagnosis of hepatic LM on the fragmented biopsy cores, in which fibrotic septa and organizing thrombus predominated; the incomplete immunohistochemical panel (PROX1 and VEGFR3 not performed at the biopsy stage) and the absence of D2-40 staining on the resection specimen preclude a definitive immunophenotypic comparison between the two specimens. Second, although the resection pathology, postoperative defervescence, normalization of inflammatory markers, and marked decline in D-dimer strongly support a causal relationship between the thrombosed hemangiomas and the recurrent fever, a definite causal relationship cannot be formally proven in a single case. Third, the duration of postoperative follow-up is still limited, as the patient is undergoing a staged treatment strategy with further procedures planned; long-term outcome and recurrence cannot yet be assessed.

## 5. Conclusions

Thrombosed giant hepatic cavernous hemangioma, which can mimic hepatic lymphatic malformation should be included in the differential diagnosis of recurrent fever with massive multifocal cystic or cystic-solid hepatic lesions, particularly when common infections are not supported and FDG uptake is low. Diagnosis should rely on integrated clinical, imaging, and pathologic assessment. Coaxial biopsy may provide valuable evidence and reduce prolonged empirical antimicrobial treatment, but its sampling limitations must be acknowledged and, when surgery is performed, the resection pathology should be used to establish the definitive diagnosis.

## Figures and Tables

**Figure 1:**
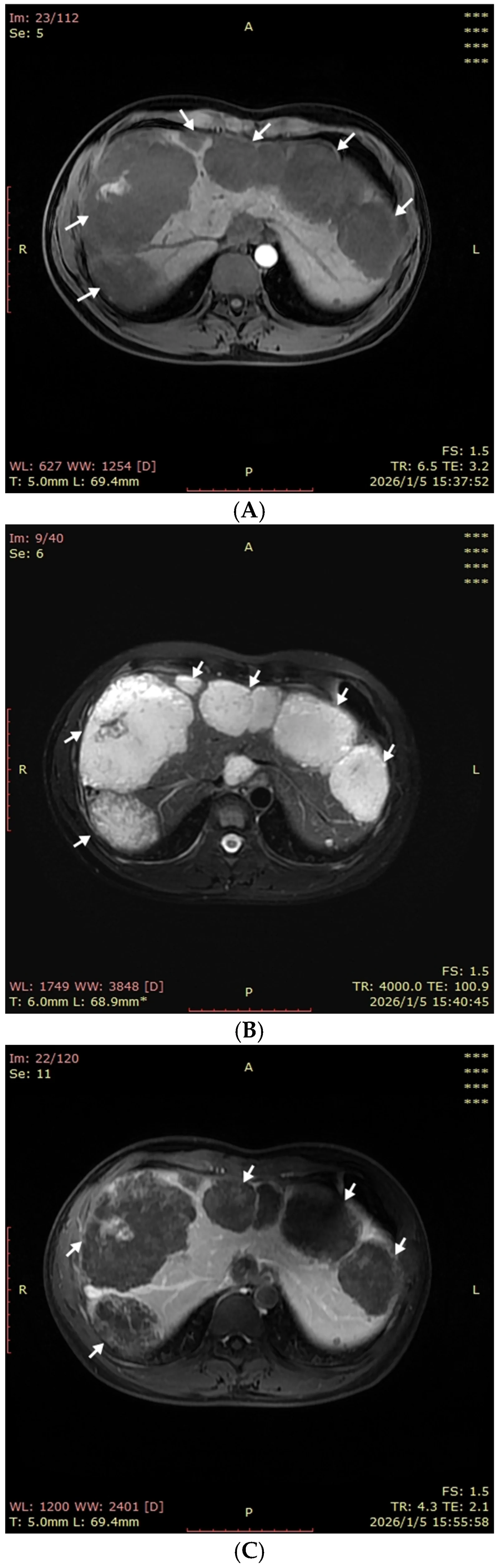

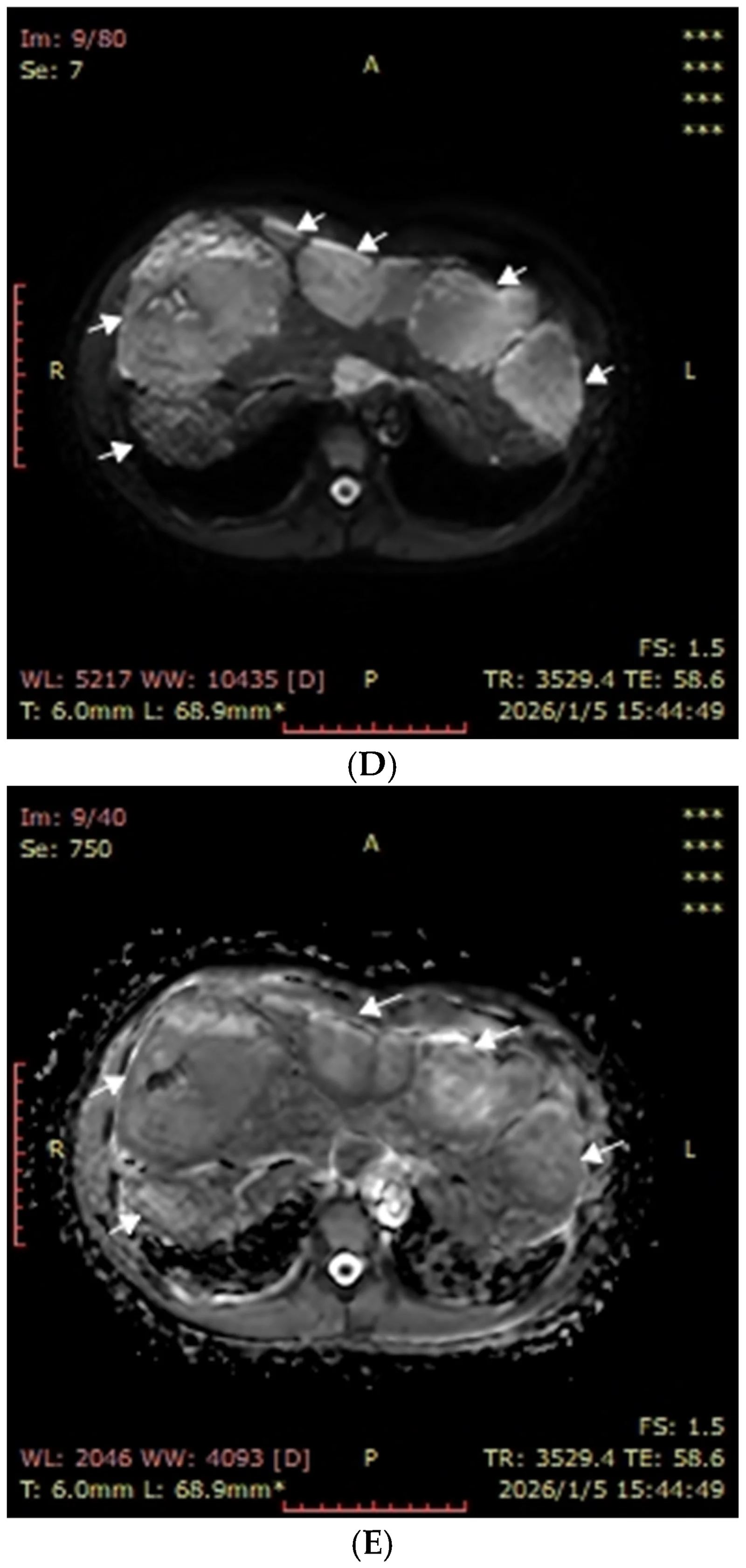
Magnetic resonance imaging of the liver. The *** indicate anonymized identifying information, and the * are scanner-generated display annotations. (**A**) Axial T1-weighted image showing marked hepatomegaly and multiple lobulated, well-defined lesions of predominantly low signal intensity in both hepatic lobes (arrows). (**B**) Axial T2-weighted image showing markedly hyperintense lesions with internal septations and multicystic morphology (arrows). (**C**) Portal-venous-phase contrast-enhanced T1-weighted image showing no appreciable enhancement of the cystic components; only thin walls and septa show mild enhancement, without a definite enhancing solid nodule (arrows). (**D**) High-b-value diffusion-weighted image showing heterogeneous high signal within the lesions without a discrete focus of marked diffusion restriction (arrows). (**E**) Corresponding apparent diffusion coefficient map showing relatively high signal/ADC within the lesions (arrows), consistent with facilitated diffusion rather than the marked restriction typically expected in a highly cellular tumor or uncomplicated pyogenic abscess.

**Figure 2:**
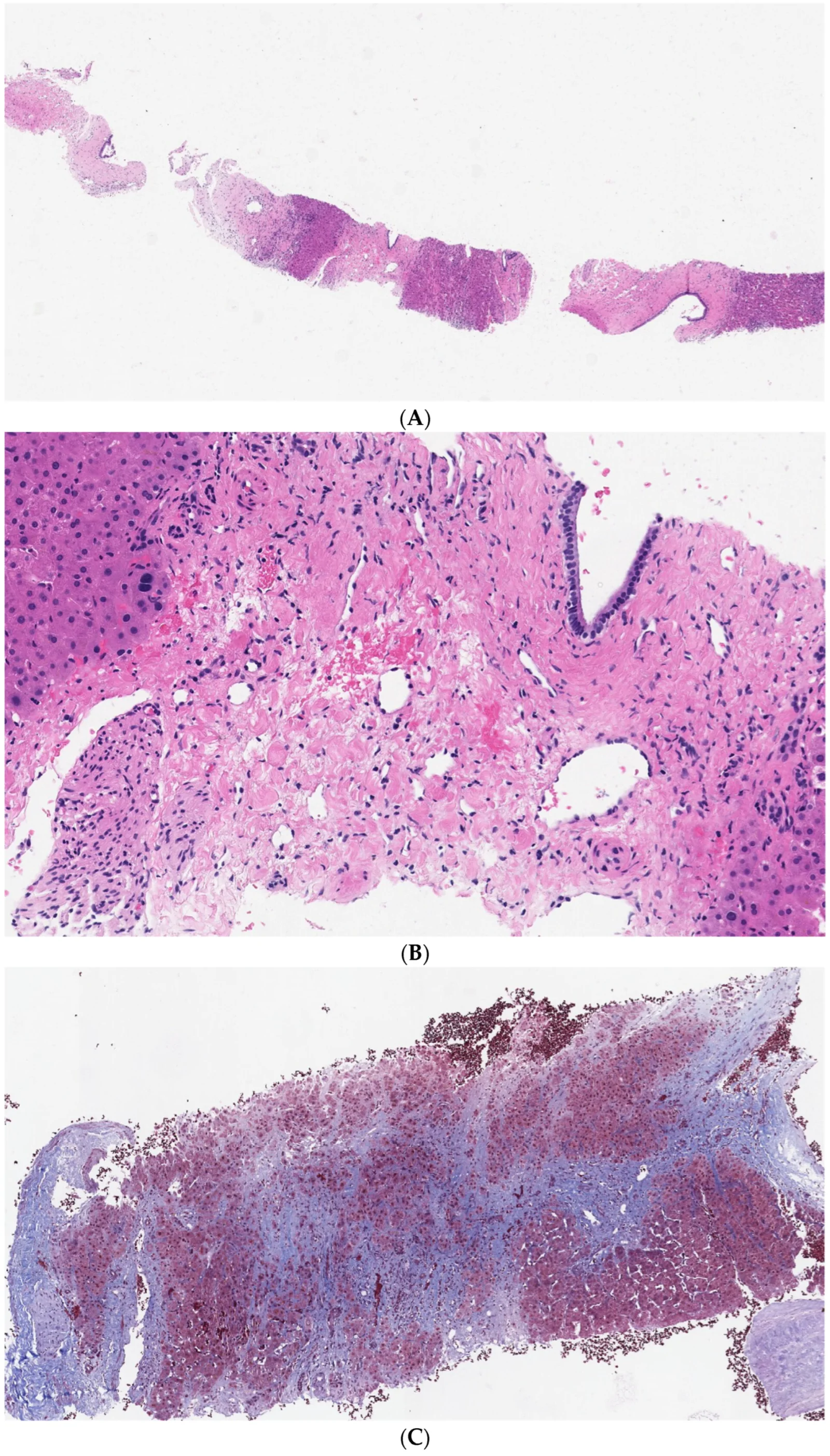
Histopathological findings of the liver coaxial biopsy. (**A**) Low-power hematoxylin-eosin view (approximately ×2) showing expanded portal areas with variably dilated vascular/lymphatic channels and surrounding stromal fibrosis, with compression of adjacent hepatic parenchyma. (**B**) Intermediate-power hematoxylin-eosin view (approximately ×16) showing thin-walled channels lined by flattened endothelial cells without cytologic atypia. Lymphoplasmacytic inflammation and ductular reaction are present in some portal tracts. (**C**) Masson trichrome stain highlighting collagen deposition around the malformed channels and within the expanded fibrotic stroma.

**Figure 3:**
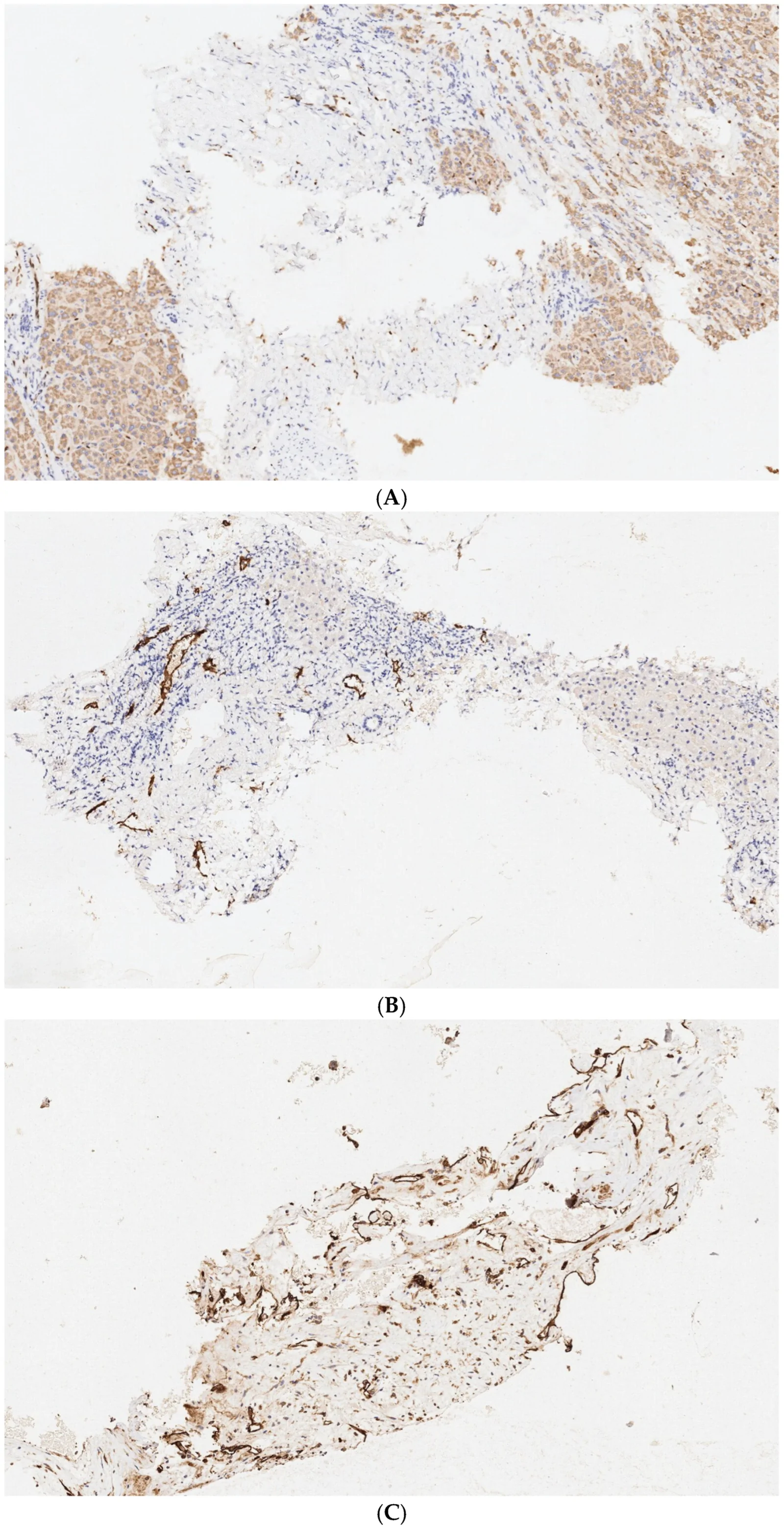
Immunohistochemical findings supporting vascular/lymphatic differentiation. (**A**) ERG immunostaining (approximately ×8–10) showing partial nuclear positivity in endothelial cells lining the dilated channels. (**B**) D2-40 (podoplanin) immunostaining (approximately ×8–10) showing partial membranous positivity in lesional endothelial cells, supporting lymphatic differentiation. (**C**) CD31 immunostaining highlighting the endothelial lining of the abnormal vascular/lymphatic channels.

**Table 1:** Clinical timeline.

Date/Period	Clinical Event	Key Findings and Management
Early October 2025	First febrile episode	Fever to 38.3 °C with dry cough; temporary improvement after azithromycin and cephalosporin therapy.
November 2025	Second febrile episode	Recurrent fever; again temporarily improved after azithromycin.
24–28 December 2025	Third episode and lesion detection	Fever to 39.8 °C; WBC 14.46 × 10^9^/L and CRP 165.30 mg/L; CT showed massive hepatomegaly and multiple calcified low-attenuation lesions.
30 December 2025	Admission	Marked, firm hepatomegaly; mildly cholestatic liver tests; D-dimer 48.44 mg/L (DDU); FDP 323.52 mg/L; platelet count, fibrinogen, PT, and aPTT normal; broad infectious and malignant evaluation initiated.
4–6 January 2026	Advanced imaging	PET/CT showed no increased FDG uptake; MRI showed multiple cystic lesions with possible calcifications and indeterminate etiology; lower-extremity venous ultrasound negative.
9 January 2026	Antibiotics stopped	Meropenem discontinued because of persistent fever, negative microbiological studies, and lack of convincing response; fever and CRP continued to decrease.
12 January 2026	Multidisciplinary review	Imaging remained indeterminate; surgery was considered high risk because of multiplicity and close apposition of lesions; biopsy recommended.
14 January 2026	Percutaneous biopsy	Ultrasound-guided biopsy (coaxial, 15-gauge needle, 5 cores, solid component of largest right-lobe lesion) with tract ablation; tissue tNGS negative; no immediate complication.
16 January 2026	Discharge	Afebrile, WBC 8.60 × 10^9^/L, CRP 63.75 mg/L; post-biopsy ultrasound showed no hematoma or subcapsular bleeding.
28 January–12 February 2026	Pathology and supplemental IHC	Report: benign vascular/lymphatic tumor, favoring hepatic lymphatic malformation.
31 March 2026	Surgical treatment (another hospital)	Laparoscopic partial hepatectomy (segments VI and IVb) combined with microwave ablation of two exophytic surface lesions; blood loss 50 mL; first stage of planned staged strategy.
April–August 2026	Resection pathology and follow-up	Resection pathology: cavernous hemangioma with thrombus formation. Patient afebrile; WBC 4.94 × 10^9^/L; D-dimer 6.95 mg/L; liver biochemistry normal. Under surveillance for planned subsequent stages.

**Table 2:** Thrombosed giant hepatic cavernous hemangioma versus hepatic lymphatic malformation.

Feature	Thrombosed Giant Hepatic Cavernous Hemangioma	Hepatic Lymphatic Malformation
Nature	Benign slow-flow vascular lesion; the most common benign hepatic tumor. Giant lesions may undergo thrombosis, infarction, sclerosis, and calcification.	Congenital lymphatic anomaly (ISSVA classification: simple lymphatic malformation; historically termed lymphangioma). Apparently isolated hepatic involvement is exceptional.
Luminal content	Blood and organizing thrombus.	Lymphatic (proteinaceous or chylous) fluid.
Histology	Dilated, thin-walled, blood-filled channels lined by bland flat endothelium, separated by fibrous septa; thrombosis, infarction, hyalinization, calcification, and cholesterol granulomas in involuting lesions.	Irregular thin-walled lymphatic channels lined by bland endothelium; stroma with lymphoid aggregates and irregular smooth-muscle bundles.
Immunohistochemistry	CD31, CD34, ERG positive; D2-40, PROX1, VEGFR3 negative. Focal or partial D2-40 staining can occur and may mislead in small biopsy samples.	CD31 and ERG positive; D2-40, PROX1, VEGFR3 positive.
Typical imaging	Classic: T2 markedly hyperintense with peripheral nodular discontinuous enhancement and centripetal fill-in. Giant or thrombosed: heterogeneous, with reduced or absent enhancement and possible calcification.	Unilocular or multilocular cysts with thin septa; septal and wall enhancement only; insinuating or trans-spatial growth.
^18^F-FDG PET	Low uptake.	Low uptake.
Coagulation	May cause localized intravascular coagulopathy with markedly elevated D-dimer and FDP; overt Kasabach–Merritt-like syndrome with thrombocytopenia and hypofibrinogenemia in extensive disease.	Usually normal; intracystic hemorrhage may occur.
Fever	Reported with intralesional necrosis, hemorrhage, or thrombosis; typically resolves after resection.	Unusual; usually incidental or mass-effect symptoms.
Coaxial-biopsy pitfalls	Sampled fibrous septa and organizing thrombus may resemble lymphatic channels; luminal blood may be washed out during processing; enhancement-poor lesions lose their diagnostic imaging pattern.	Small samples may not capture diagnostic architecture; full lymphatic-marker panel (D2-40, PROX1, VEGFR3) required for confident diagnosis.
Treatment	Observation for asymptomatic lesions; enucleation or resection for symptomatic, growing, or uncertain lesions; embolization; staged resection with or without thermal ablation for multifocal disease; transplantation in exceptional diffuse cases.	Observation for selected asymptomatic lesions; complete resection when feasible; sclerotherapy for macrocystic disease; sirolimus for refractory or diffuse disease; transplantation in exceptional cases.

Note: Table S1 (radiological differential diagnosis of multifocal cystic or cystic-solid hepatic lesions) and Table S2 (reported adult cases of giant hepatic cavernous hemangioma presenting with fever, marked inflammation, or Kasabach–Merritt-like coagulopathy) are provided in the accompanying Supplementary Tables File due to their width.

## Data Availability

The data supporting the findings of this case report are contained within the article. Additional data are not publicly available due to patient privacy and ethical restrictions.

## Supplementary Materials

The following supporting information can be downloaded at: https://www.mdpi.com/article/10.3390/jcm15197366/s1, Table S1. Radiological differential diagnosis of multifocal cystic or cystic-solid hepatic lesions. Table S2. Reported adult cases of giant hepatic cavernous hemangioma presenting with fever, marked inflammation, or Kasabach-Merritt-like coagulopathy, and the present case.
