# Supplementary material for: Thrombosed Giant Multifocal Hepatic Cavernous Hemangioma Mimicking Lymphatic Malformation on Coaxial Biopsy in an Adult with Recurrent Fever: A Case Report and Focused Literature Review
Source: J Clin Med. 2026 Sep 22;15(19):7366. doi: 10.3390/jcm15197366 (PMC13665710; doi:10.3390/jcm15197366)
Supplement: Supplementary file 1 [file jcm-15-07366-s001.zip › jcm-4472939-supplementary.pdf]

Table S1. Radiological differential diagnosis of multifocal cystic or cystic-solid hepatic lesions.

Entity	CT	MRI (T1 / T2)	Enhancement pattern	DWI / ADC	18F-FDG PET	Key distinguishing features
Hepatic lymphatic malformation	Well-defined hypoattenuating unilocular or multilocular cysts with thin septa; calcification may occur; may insinuate around vessels or extend exophytically	T1 low (high if chylous or hemorrhagic content); T2 markedly high with septations	Thin wall and septal enhancement only; no enhancing solid nodule; no blood-pool pattern	Facilitated diffusion; high ADC	Low	Lymph-containing spaces; trans-spatial or insinuating growth; septal-only enhancement
Cavernous hemangioma (classic)	Well-defined hypoattenuating lesion	T1 low; T2 markedly high (light-bulb sign)	Peripheral, nodular, discontinuous enhancement with centripetal fill-in, paralleling the blood pool	No true restriction (T2 shine-through)	Low	Blood-pool enhancement pattern is virtually diagnostic
Giant, thrombosed, or sclerosed hemangioma	Heterogeneous hypoattenuation; central scar-like areas; punctate or coarse calcification possible; exophytic growth in giant lesions	Heterogeneous T2; thrombosed or fibrotic areas of low signal within high-signal lakes	Peripheral nodular enhancement may be minimal, patchy, or absent; fill-in incomplete or lost	Variable; no discrete marked restriction	Low	May closely mimic lymphatic malformation or cystic neoplasm, as in the present case; thrombosis, fibrosis, and calcification obscure the classic pattern
Pyogenic abscess	Low-attenuation lesion with enhancing rim; gas possible; perilesional edema	T1 low; T2 high center with perilesional hyperintensity	Rim enhancement; double-target sign	Marked central diffusion restriction (low ADC)	High	Sepsis, response to antibiotics or drainage; restricted central diffusion
Hepatic echinococcosis	Cyst with daughter cysts, detached membranes (water-lily sign), curvilinear wall calcification	T1 low; T2 high with characteristic low-signal rim (pericyst)	Wall and septal enhancement; no enhancing nodule	Variable	Usually low unless complicated	Endemic exposure; positive serology; daughter cysts and membranes
Mucinous cystic neoplasm	Solitary multilocular cyst with septa, usually left lobe; wall nodule possible	T1 variable (mucin); T2 high	Septal and wall enhancement; enhancing mural nodule suggests malignancy	Variable	Low to moderate	Middle-aged women; solitary; ovarian-type stroma on pathology
Cystic metastasis	Multiple lesions with irregular, thick walls; primary tumor often known	T1 low; T2 intermediate to high	Irregular rim enhancement	Restricted diffusion in viable rim	Usually high	Known primary (e.g., GIST, neuroendocrine, mucinous colorectal, ovarian)

Epithelioid hemangioendothelioma	Peripheral coalescent nodules; capsular retraction; lollipop sign (occluded vein at lesion edge)	T2 target appearance	Halo or target enhancement	Variable restriction	Variable, often moderate	Young to middle-aged adults; capsular retraction; CAMTA1 or TFE3 rearrangement on pathology

Abbreviations: ADC, apparent diffusion coefficient; CT, computed tomography; DWI, diffusion-weighted imaging; FDG, fluorodeoxyglucose; LM, lymphatic malformation; MRI, magnetic resonance imaging. In the present case, extensive thrombosis, fibrosis, and calcification abolished the classic hemangioma enhancement pattern; resection pathology established the diagnosis.

Table S2. Reported adult cases of giant hepatic cavernous hemangioma presenting with fever, marked inflammation, or Kasabach-Merritt-like coagulopathy, and the present case.

Author, year	Age / Sex	Presentation	Lesion(s)	Key laboratory findings	Pathology	Treatment	Outcome
Lee et al., 1994 (J Korean Med Sci)	NA	Fever of unknown origin	Giant hepatic hemangioma	NA	Cavernous hemangioma	NA	NA
Aslan et al., 2009 (Case Rep Gastroenterol)	44 / F	Kasabach-Merritt syndrome	Giant hepatic hemangioma	Thrombocytopenia; consumptive coagulopathy	Cavernous hemangioma	Enucleation	Coagulopathy resolved; resection usually curative
Wang et al., 2020 (J Int Med Res)	59 / F	Fever up to 40 °C for 6 months, cough, night sweats, weight loss	Solitary, right lobe, 10 × 9 cm	WBC 15.7 × 10^9/L; CRP 40 mg/L; ESR 90 mm/h; anemia (Hb 87-104 g/L)	Cavernous hemangioma with hemorrhage, necrosis, and inflammatory infiltration	Emergency right-lobe hemangioma resection	Fever resolved postoperatively; no complications
Nguyen et al., 2024 (ACS Case Reviews in Surgery)	42 / M	Enlarging giant hemangioma with subclinical coagulopathy	Solitary, up to 25.2 cm (grew from 16 cm over 9 years)	Subclinical Kasabach-Merritt phenomenon: platelets 94-130 × 10^9/L; mildly prolonged PT; mild anemia	Cavernous hemangioma	Preoperative arterial embolization, then resection	Coagulopathy resolved; platelets normal at 7 months
Deng et al., 2025 (HepatoBiliary Surg Nutr)	51 / F	Abdominal pain and distension, jaundice, petechiae; Kasabach-Merritt syndrome	Right liver, 28.7 cm (73% of liver volume)	Platelets 73 × 10^9/L; fibrinogen 0.79 g/L; D-dimer 2.88 mg/L; FDP 28.6 mg/L	Cavernous hemangioma	Staged: TACE × 4, then mesohepatic resection	Coagulation normalized; no recurrence at 6 months
Ryozawa et al., 2025 (Clin J Gastroenterol)	40 / F	Postpartum (9 days) fever of unknown origin (38.3 °C), fatigue	Right posterior lobe, 13 cm	CRP 103.6 mg/L; IL-6 47.6 pg/mL; mild anemia (Hb 89 g/L)	Cavernous hemangioma with coagulative necrosis (infarction) and neutrophil infiltration	Hepatic resection	Fever resolved; CRP and IL-6 normalized

Present case, 2026	32 / M	Recurrent fever for >2 months (max 39.8 °C), massive hepatomegaly	Multifocal, bilobar; largest ~11 cm, exophytic; punctate and coarse calcifications	WBC 14.46 × 10^9/L; CRP 165.3 mg/L; IL-6 35.1 pg/mL; D-dimer 48.44 mg/L; FDP 323.5 mg/L; platelets and fibrinogen normal	Core biopsy favored lymphatic malformation; resection: cavernous hemangioma with thrombosis, infarction, calcification, cholesterol granulomas	Staged: laparoscopic resection of dominant lesions (S6, S4b) plus microwave ablation of surface lesions; further stages planned	Afebrile since surgery; WBC normal; D-dimer decreased to 6.95 mg/L; under surveillance

Abbreviations: aPTT, activated partial thromboplastin time; CRP, C-reactive protein; ESR, erythrocyte sedimentation rate; FDP, fibrin degradation products; IL-6, interleukin-6; LM, lymphatic malformation; PT, prothrombin time; TACE, transarterial chemoembolization; WBC, white blood cell count.
